# Meeting of the Physicians of Whitesides County, Illinois

**Published:** 1851-11

**Authors:** A. Smith, H. C. Donaldson

**Affiliations:** President; Como, Ills.; Secretary, pro tem.; Como, Ills.


### ARTICLE VI.

### *Meeting of the Physicians of Whiteside County, Illinois.*

Como, Ills., Sept. 27th, 1851.

Pursuant to previous arrangement the Physicians of Whiteside County met at Union Grove, July 22d, 1851, for the purpose of organizing a County Medical Association, Doct. A. Smith, Prest. pro tem and Doct. H. C. Donaldson, Sec'y.

Committee to draft Constitution and By-Laws reported, which report after some amendments was adopted.

On motion of Dr. Abbott the code of ethics adopted by the Rock River Medical Society was adopted by the Whiteside County Medical Society.

On motion, the Society proceeded to the election of Offi cers for the succeeding year which resulted as follows:

Dr. A. Smith, of Lyndon, President.
" J. C. Bardwell, of Prophetstown, Vice President.
" A. G. Porter, of Como, Secretary.
" H. C. Donaldson, of Lyndon, Treasurer.

*Board of Censors*—Doct's. Hudson, Abbott and Donaldson.

President appointed Dr. Abbott to read an original Essay before the Society at its next meeting. The annual meeting of this Society is on the first Tuesday in each June, and the semi-annual meeting on the first Tuesday in December.

The following Resolutions were unanimously adopted, viz:

*Resolved*, That should any individual refuse to pay the just bill of his Physician by taking refuge behind the late Homestead Ex-Emption Act, it shall be just and proper for such Physician to refuse further attendance until payment or satisfaction is rendered.

*Resolved*, That any neighboring Physician on being duly informed of such refusal to pay, shall decline any attendance until the first Physician is paid or his debt secured.

*Resolved*, That the North-Western Medical and Surgical Journal be furnished a copy of the above for publication; also, the Chicago Democrat, Chicago Tribune and the Chicago Journal.

On motion, the Society adjourned to meet at Lyndon on the first Tuesday in December, 1851.

A. SMITH, *President.*

H. C. DONALDSON, *Secretary, pro tem.*

